# A Novel Technique to Increase the Thickness of TiO₂ of Dental Implants by Nd: DPSS Q-sw Laser Treatment

**DOI:** 10.3390/ma13184178

**Published:** 2020-09-20

**Authors:** Antonio Scarano, Francesca Postiglione, Ahmad G. A. Khater, Faez Saleh Al-Hamed, Felice Lorusso

**Affiliations:** 1 Department of Medical, Oral and Biotechnological Sciences and CeSi-MeT, University of Chieti-Pescara, Via dei Vestini, 31, 66100 Chieti, Italy; felice.lorusso@unich.it; 2 Department of Medical, Oral and Biotechnological Sciences and CasT, University of Chieti-Pescara, Via dei Vestini, 31, 66100 Chieti, Italy; francesca-postiglione@hotmail.com; 3 Faculty of Oral and Dental Medicine, Ahram Canadian University, Giza 12511, Egypt; 6141598.ahmed@acu.edu.eg; 4 Faculty of Dentistry, McGill University, Montreal, QC H3A 1G1, Canada; Faez.al-hamed@mail.mcgill.ca

**Keywords:** TiO_2_, laser surface, implant surface, hydrophilic surface, bone healing

## Abstract

High bone–implant contact is a crucial factor in the achievement of osseointegration and long time clinical success of dental implants. Micro, nano, microtopography, and oxide layer of dental implants influence tissue response. The lasers were used for achieving an implant surface with homogeneous micro texturing and uncontaminated surface. The present study aimed to characterize the implant surfaces treated by Nd: DPSS Q-sw Laser treatment compared to machined implants. A total of 10 machined implants and 10 lasered surface implants were evaluated in this study. The implant surfaces were evaluated by X-ray Photoelectron Spectroscopy (XPS), Auger Electron Spectroscopy (AES), and metallography to characterize and measure the thickness of the oxide layer on the implant titanium surface. The machined surfaces showed a non-homogeneous oxide layer ranging between 20 and 30 nm. The lasered implant surfaces showed a homogeneous oxide layer ranging between 400 nm and 460 nm in the area of the laser holes, while outside the layer, thickness ranged between 200 nm and 400 nm without microcracks or evidence of damage. Another exciting result after this laser treatment is a topographically controlled, repeatable, homogeneous, and clean surface. This technique can obtain the implant surface without leaving residues of foreign substances on it. The study results indicate that the use of Nd: DPSS Q-sw laser produces a predictable and reproducible treatment able to improve the titanium oxide layer on the dental implant surface.

## 1. Introduction

High bone density in contact with the implant is a crucial factor in the achievement of osseointegration and clinical success of dental implants for oral fixed implant rehabilitation [1]. Micro, nano, and macrotopography of dental implants influence tissue response [2], and there is a great interest in the properties of implant surfaces [3]. Several authors have investigated the influence of physicochemical composition and nano and micro-topography, which have a significant impact on the outcome of osseointegration [4]. There is potentially an optimal microroughness affecting the initial healing processes [5] and bone regeneration procedure [6]. This great interest focuses on utilizing surface-modification treatments with the intent to shorten the healing times and augmentation life of dental implants. In fact, modifying the roughness of the implant surface, using different techniques, improves the bone–implant contact and biocompatibility and cell viability. The roughness affects the bone reaction and provides a better bone response when it is 1.5 μm larger than the machined surfaces [7]. Different methods are used to roughen the surface of titanium implants and they are categorized based on whether addition or subtraction into chemical, mechanical, and physical methods. Sandblasting with abrasives on AlO_2_, HA, TiO_2_, laser, acid-etching, or dual acid-etching, electrochemical deposition, or combining of these techniques are subtractive methods. Addition techniques can be used such as plasma spray, bioactive ceramic incorporation, calcium phosphate (CaP), hydroxyapatite (HA), and polymer crystal coatings or polyetheretherketone (PEEK) coating. Also, the coating on dental implants of the critical components of the extracellular matrix, enzymes, peptides, or collagen type I were used to enhancing cellular response. These surfaces improve and facilitate osseointegration and confer a very high survival rate, 95.7% and 92.8% respectively after 5 and 10 years of follow-up in healthy patients. Another approach involves the modification of the topographical and physico-chemical characteristics of implant surfaces. Hydrophobicity and roughness of implant surfaces are considered to play a significant role in helping in vivo osteoblasts to promote osseointegration. For this reason, achieving implant surfaces with homogeneous micro- or nano-topography could be a promising approach, for surfaces with good biological properties.

After the production cycle, a titanium dioxide (TiO_2_) layer, with a thickness of 3–7 nm, naturally forms on the titanium implant surface [8]. This layer passively reduces metallic ion release from the titanium implant to the adjacent tissues [9], avoiding undesirable body reactions [10] and is of great importance for its biocompatibility and successful osseointegration. Consequently, various surface oxidation techniques were used to alter the surface properties of the natural passive layer to improve osseointegration. As the thickness of the titanium oxide layer increased to 100~150 nm, there was a higher wettability with a positive function in promoting pre-osteoblast activity [11]. This TiO_2_ layer incorporates P and Ca ions into the surface layer [12].

Lasers were used to achieve an implant surface with a homogeneous micro texture, and studies in-vivo and in-vitro demonstrated a high degree of biocompatibility [13]. The laser treatment of titanium surfaces has brought about many advances in the modulating of the gun parameters and cutting precision at the micro-level. This technique helps to obtain a clean and reproducible micropattern on implant surfaces [14]. Previously, in vivo and in vitro studies showed that laser treatment improves wettability [15], reduces bacterial adhesion [14], and improves osseointegration [16].

This study aimed to characterize the implant lasered surfaces by XPS, AES, and metallography, then to evaluate the thickness of titanium oxide on the surface.

## 2. Materials and Methods

### 2.1. Surface Treatment

In this study, we used a threaded commercial titanium grade IV with α structure (Way Syntegra implants, Geass, Pozzuolo del Friuli, Udine, Italy) 3.8 mm in diameter and 11 mm in length.

The implants were divided as follows: 10 implants with machined surfaces (M) and 10 implants with lasered surfaces, SYNTEGRA^®^ (L) (Geass, Pozzuolo del Friuli UD, Italy). Test implants were developed using laser technology. The neodymium laser with Nd: DPSS Q-sw, which is a diode-pumped solid-state laser (Geass, Pozzuolo del Friuli UD, Italy), with a neodymium source, operating in Q-switching mode. This laser produces pulses of short duration (some tens of nanoseconds) and high-level peak power (some tens of megawatts). This laser, with a wavelength of 355 nm, was set to produce a pulse frequency of 5 kHz, with a pulse duration of 20 ns. The laser produces high-intensity, high-brightness pulses and which are targeted for titanium micro ablation. The laser treatment produces a macro-concavity with a size of 20 µm diameter with 30 μm pitch and a hemispheric pore depth of 5 μm.

### 2.2. XPS (X-ray Photoelectron Spectroscopy)

Ten titanium laser textures and ten controls were evaluated to chemically characterize the implant surface composition using the X-ray Photoelectron Spectroscopy. The samples were composed of titanium grade 4, characterized by a size of 3.8 mm diameter and 11 mm in length. X-ray Photoelectron Spectroscopy characterization has been done using a Perkin Elmer PHI 5600 ESCA instrument (PerkinElmer Inc., Waltham, MA, USA). The device has a monochromatic Al anode with a parameter setting of 10.1 kV and 200 W. The measured spot length was about 500 μm, while the evaluated depth approx. 8 ns. The base pressure was preserved at 10 Pa. The angle parameter between the electron analyzer and the experimental implant surface was set at 45°. The assessment was carried out by a wide-range survey spectra acquisition (0–1000 eV binding energy) according to the following parameters of pass energy of 117.4 eV, resolution of 1 eV/Step, and acquisition time of 40 ms/Step. After the assessment of the analysis spectra, high-resolution C1s peaks were evaluated according to pass energy of 11.75 eV, resolution of 0.100 eV/Step, and acquisition time of 50 ms/Step. The deconvolution of C1s peak was performed in the integrated device software package, according to a rectification of the peak position with a quotation to the regular internal C–C component of the C1s peak positioned at 285.0 eV.

### 2.3. Auger Electron Spectroscopy (AES)

Ion-Beam Dual-Beam Strata DB235 (FIB/SEM) rasters focus energetic beams of electrons and Ar^+^ ions for removal of material from implants. This instrument uses the energetic ion beam to remove thin amounts of material of less than 100 nm thick, for analysis using a transmission electron microscope (JEOL Ltd., Tokyo, Japan). Using a 3 keV energy electron source, ionic erosion of the surface was carried out using Ar^+^ ions. The mean depth of analysis for an AES measurement is approximately 5 nm. For lasered implants, the analysis was carried out both in a hole and outside it.

### 2.4. Focused Ion Beam Scanning Electron Microscopes (SEM/FIB)

Focused Ion Beam Scanning Electron Microscopes combines the 3D imaging and analysis performance of the FIB (Dual Beam FEI STRATA DB 235, Hillsboro, OR, USA) with both a focused (Gallium) Ga^+^ ion beam-column and a high-resolution field emission scanning electron. Precise three-dimensional imaging was taken, particularly of microscopic details of holes. Scanning electron microscopy with a focused ion beam was used to examine particularly fine details, such as the areas of passage between the bottom and the outer edge of the holes. The ion beam was used to carry out excavations to investigate the sample in depth without damaging its surface. This technology cutting/milling technique uses a dense beam of Ga^+^ ions to mill deep areas adjacent to the area of interest, thereby allowing the exhumation of a small portion of the sample and depositing a thin platinum film. Subsequently, the electronic column is used to perform electron microscopy images within the excavation. The cross-section shows descriptions of the surface formed during the first stage of ion milling and the titanium grains configuration in two dimensions. The roughness measurements were achieved using the software that allows transforming conventional SEM images into three-dimensional data (Alicona Imaging GmbH, Grambach, Graz, Austria). Four areas for obtaining an image size of 150 µm^2^ were randomly selected on the implant surface, and the roughness surface was calculated as the roughness Ra parameter.

### 2.5. Metallography

The experimental samples were treated in order to obtain thin ground sections by Precise 1 Automated System (Assing, Rome, Italy). The obtained specimens were embedded into a high hard resin (Technovit 7200 VLC, Kulzer, Wehrheim, Germany). At the end of the polymerization process, dental implants were split along their major axis with a high-precision disc-shaped at approximately 130 μm and ground to approximately 30 μm using a dedicated custom grinding instrument. A total of 4 sections were obtained for each implant specimen. For metallographic analysis, an acid attack on the lapped and determination surface was used for metallographic structure evaluation. The samples were immersed in an acid solution: Keller (2 mL HF, 3 mL HCl, 5 mL HNO_3_, 190 mL H_2_O). The attack was carried out for the laser samples for 20 s and 2 min for machined implants. The images acquired were analyzed by a light stereomicroscope connected to a video camera with a high-resolution (16.25-mpx) (Digital Sight series microscope Nikon, Minato-ku, Tokyo, Japan), a high-resolution screen, and a personal computer (TOSHIBA PORTÉGÉ X30T-E-113, Tokyo, Japan). The optical device was connected to a dedicated metric software package capable of acquiring images captured by a digital camera (Sony α330, Nihonbashi, Tokyo, Japan) and subjected to morphometric evaluation by digital image-evaluation (NIS-Elements AR 3.0 software, Nikon, Minato-ku, Tokyo, Japan).

### 2.6. Statistical Evaluation

The sample size was calculated by the power analysis performed using dedicated software, freely available on the website http://clincalc.com/stats/samplesize.aspx, to determine the number of experimental samples for each group necessary to obtain a statistically significant quantitative measurement of morphometry. For dichotomous variables (yes/no effect) a measurement model was embraced by leaving the effect incidence designed to caution the reasons as follow; controls (15%), laser-treated (85%), whereas alfa set at 0.05 and 90%. The optimal quantity of experimental specimens for the study was 16 implants. The normal distribution of the study data was tested by the Shaphiro Wilks test. The differences between the study groups of implants were measured by the Student test. A *p*-value < 0.05 was considered statistically significant. The study data were statistically analyzed by the software package GraphPad 6 (Prism, San Diego, CA, USA).

## 3. Results

### 3.1. XPS (X-ray Photoelectron Spectroscopy)

Samples in the control group were just machined. The X-ray Photoelectron Spectroscopy detected implant surface characterization of machined and laser texture surfaces samples are presented in Table 1. Temporarily, the surface of titanium appears coated by a thin oxide layer of around 20 nm; thus, the highest theoretical concentration of Ti on pure titanium is 33%, while the other major component is oxygen (the most stable oxide is TiO_2_).

**Figure 1:**
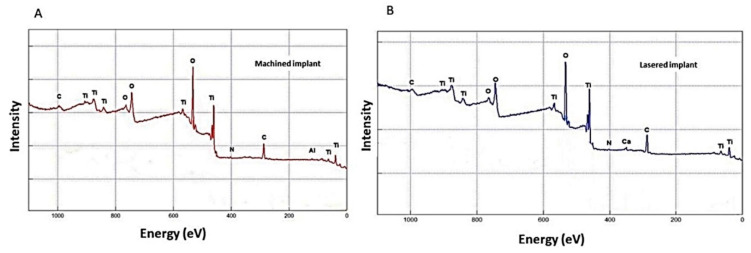
(**A**) XPS spectra of the implant surface of machined samples. The prominent photoemission peaks of the different elements present on the surface are highlighted. (**B**) XPS spectra of the implant surface of laser texture samples. The main photoemission peaks of the different elements present on the surface are highlighted.

The hydrocarbons from the atmosphere are ubiquitous and adsorbed then contamination of the surface produces a carbon surface overlayer, readily detected by surface-sensitive techniques such as X-ray Photoelectron Spectroscopy, where a decrease of the Ti concentration under the theoretical value is evident. No calcium, sodium, chlorine, sulfur, were detected (Table 1, Figure 1). The lasered implants presented a significant concentration of calcium, silicon, and a minor concentration of nitrogen, while no chlorine, sulfur, and aluminum were detected (Table 1 and Figure 1).

### 3.2. Auger Electron Spectroscopy (AES)

**Figure 2:**
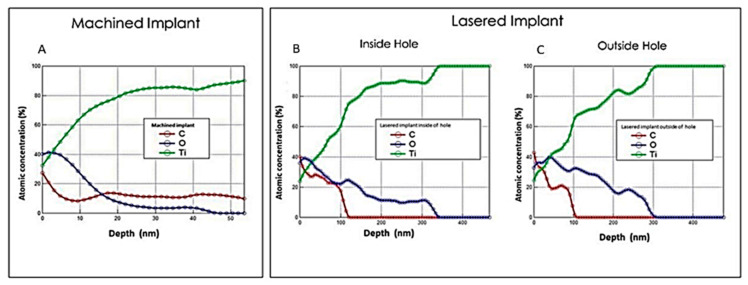
(**A**) concentration profile with varying depth for the machined implant. Concentration profile with varying depth for the Lasered implant. (**B**) Inside a hole. (**C**) Outside a hole.

The sectional images of the machined samples show the surface oxide layer, since it has a chemical nature similar to that of the underlying material, and also because of its small thickness, it is difficult to obtain images that allow accurate measurement. In the machined implants, oxide layers were observed with thicknesses between 30–50 nm, even if already around 12–15 nm (mean: 40.5 ± 9.5 nm), the percentage of oxidized titanium is minimal in all surfaces of the implants (Figure 2). While in the lasered implant inside the hole produced by the laser, a uniform surface layer oxidation with a thickness of between 300 nm and 400 nm (mean: 350 ± 100.1 nm) can be observed. On the outside of the hole, however, a uniform oxidation layer is not observed, since it forms islands of between 200 nm and 400 nm thick (mean: 300 ± 101.2 nm), lower than previously observed inside the hole (Figure 2).

### 3.3. Focussed Ion Beam Scanning Electron Microscopes (SEM/FIB)

**Figure 3:**
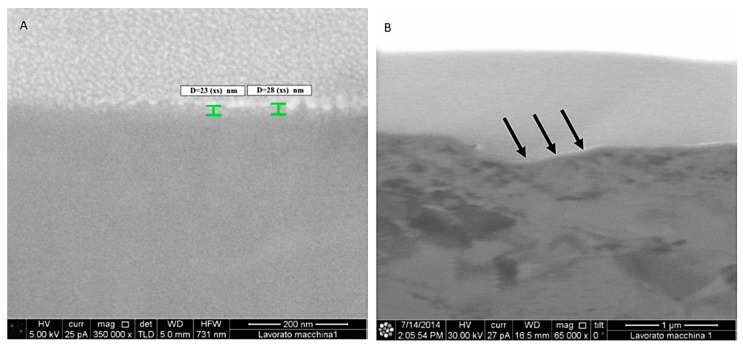
(**A**) Thickness of the oxidation layer of a machined implant. (**B**) Thickness of the oxidation layer (arrows).

**Figure 4:**
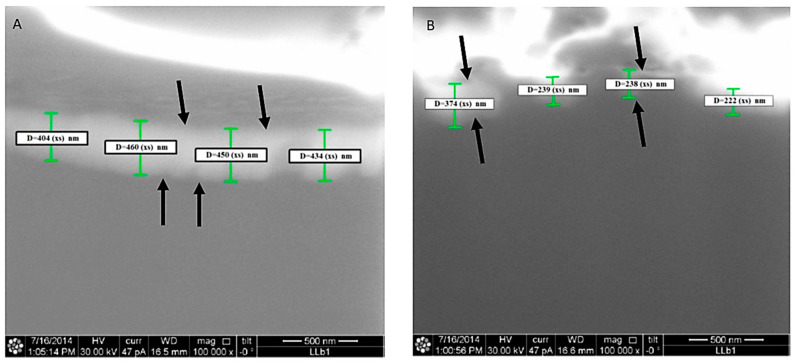
Thickness oxidation layer of a lasered implant (Arrows). (**A**) Inside a hole. (**B**) Outside a hole.

**Figure 5:**
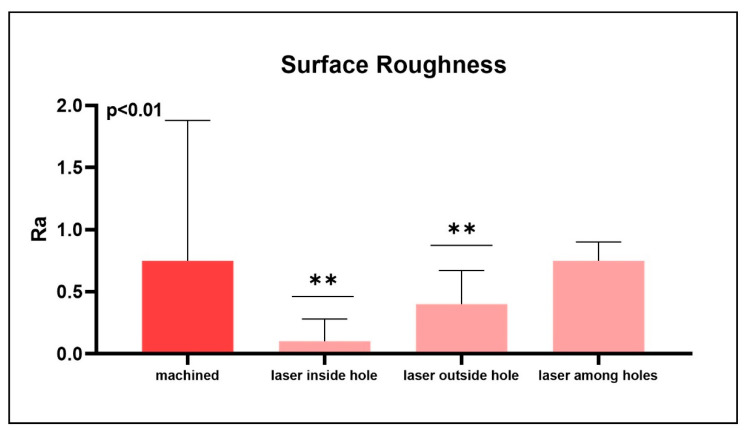
Surface roughness of the machined implants and laser treatment inside the holes generated outside the holes and among the holes (** *p* < 0.01).

**Figure 6:**
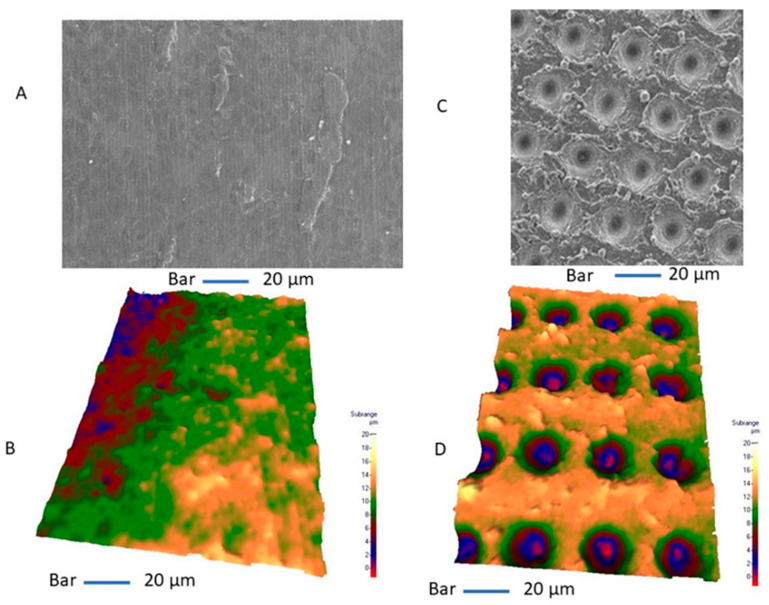
Conventional SEM image transformed into three-dimensional data for evaluation of surface roughness. (**A**,**B**) Machined implant. (**C**,**D**) Lasered implant. The laser treatment produces a macro-concavity with a size of 20 µm diameter with 30 μm pitch and a hemispheric pore depth of 5 μm.

The studied titanium shows sharply defined areas of electron diffraction patterns corresponding to the titanium structure. In the machined implants, oxide layers with a thickness between 20 and 30 nm (Figure 3) were observed. While in the lasered implants, inside the hole produced by the laser, a uniform surface layer oxidation with a thickness of between 400 nm and 460 nm can be observed by ion microscopy (Figure 4). On the outside of the hole, however, a uniform oxidation layer is not observed, since it forms islands between 200 nm and 400 nm thick, lower than previously observed inside the hole. The Ra average was 0.75 µm for machined implants (mean: 0.75 ± 0.13 µm) while for lasered implants it was 0.1 µm inside the hole (mean: 0.1 ± 0.18 µm), 0.4 µm outside of the hole (mean: 0.4 ± 0.27 µm) and 0.75 µm between the holes (mean: 0.75 ± 0.15 µm) (Figure 5 and Figure 6).

### 3.4. Metallography

**Figure 7:**
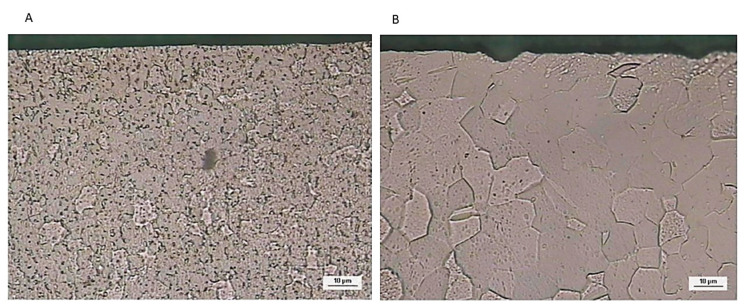
The grain sizes of the machined implant (**A**) and lasered implant (**B**).

The grain boundaries of the implant titanium specimens, observed at 200× magnification, sometimes appearing gray, sometimes a lighter dark if compared to the grains themselves. The implants treated with laser show grain size larger than the machined implants. The average grain sizes of the machined implants were 6 ± 1.3 µ, while in lasered implants, they were 13 ± 1.5 µ (Figure 7).

## 4. Discussion

In the present study, it was found that the laser Nd: DPSS Q-sw removes the titanium through sublimation, moving on the surface of the implant and creating thousands of micrometrical pores regularly distributed and the same size and shape and increases the TiO_2_ thickness. The result after this laser treatment is a topographically controlled, repeatable, homogeneous, and clean surface. This technique can obtain the surface without leaving residues of foreign substances on the implant itself.

In the present research, we found aluminum on the machined surfaces, probably a residue from the mechanical processing, while for the lasered samples, there was the presence of calcium. Calcium is a common surface contaminant, possibly related to the laser processing of samples. Aluminum is an undesirable element suspected to reduce bone formation through a potential action competitive to calcium. However, the data in the literature are not sufficient to draw conclusions [17,18,19]. Direct laser ablation or direct etching laser, by means of a focused laser beam, is a method for achieving a clean and precise sandblast to the implant surface. Precision is, therefore, mainly dependent upon the main laser parameters, peak power, such as spot size and pulse duration. DPSS lasers can treat the implant surface with high precision. With this technique, a thin titanium layer is removed from the surface in the form of gas, and this ablation ensures clean processing with thermally altered areas, without microcrack formation, and with high repeatability of the process. A thermal modification has been observed in the present study; in fact, the grains size has a major dimension in the implant surface treated by laser. Laser high-energy light interacts with the surface metal, producing either ionization of a small quantity on the surface or both ionization and melting, that are related to the laser type peak power, such as spot size and pulse duration. During this interesting phenomenon, the microablation ablated titanium is evaporated outside from the back of the holes; hence it is possible to sandblast in-depth with the homogeneous diameters. Dented surface will be produced in Ti surface for the areas where laser is shone, while the other areas remains intact. Laser treatment is an ablative technique; for this reason, it is essential to control the laser power or shining time to regulate the amount of materials removed by laser; otherwise, the Ti will have an uneven surface. This technique has been used in electronic engineering [20] and dental implants [21]. The surface treated with Nd: DPSS Q-sw shows a high magnification micrograph of an implant surface sandblasted with laser etching. The width of the holes is very homogeneous, and they have steep edges and are clean with cavities of 20 μm diameter and 30 μm with interpore distance. The laser treatment decreases the surface roughness inside and outside the holes, but the macro-geometry and a greater thickness of the TiO_2_ layer enhance osteogenic activity [11] and reduce bacterial biofilm [14].

The laser treatment of the surface is a process with high repeatability that avoids any contamination of the surface by other elements, without cluster formation and with a few microstructural modifications. Therefore, we have found a formation of grain sizes after laser treatment, probably due to a fusion of grains. The laser treatment creates detailed textures on implant surfaces, giving them specific properties and improving biological response and quality, and patient safety. In fact, in a previous study, we found that this treatment modifies the surface energy and increases the wettability and extension of the fibrin clot attachment on titanium, having a direct effect on protein adsorption [15]. In the first stage of bone healing, these biological events probably increase the osseointegration process [22]. Today, great attention is directed on surface-modification treatments for shortening healing times and increasing implant duration. The approaches which have been found that increase bone–implant contact includes micromorphology, chemical properties [23], and micro coating, such as calcium- and phosphate-based coatings on Ti surfaces, which further enhances bone formation through the lonely texture of Ti surfaces [24]. The surface microstructure and surface chemistry, such as thin depositions of calcium phosphate (CaP) crystals and hydroxyapatite (HA) on implant surfaces, increase healing and improve the strength of the bond between the implant and bone [25]. Appropriate implant surface roughness is sought to promote cell adhesion and bone neoformation and is the current trend in implantology. It has been shown that micro and nanosurfaces are able to stimulate a higher osteoblastic activity and, therefore, the dental implant osseointegration. For these reasons, many studies have shown an increase in bone–implant contact, especially in the first phase of bone healing on surfaces with higher roughness. The disadvantage of roughness is the difficulty of removing surface contaminants, and this can cause a tissue reaction. Traces of the lubricants, materials used, such as metals, detergents, and metal ions, can be detected on the implant surfaces, and elimination of adverse chemical compounds must be done to improve implant tissue response [26]. Other proposed strategies to improve implant-bone contact include increasing TiO_2_ layers on the surface [27], and in this study, we found that laser treatment increases the thickness of TiO_2_. Laser treatment can create an implant surface with a repeatable structure, allowing for biological response and a good vascularization and osseointegration. Laser treatment proved to increase TiO_2_ layers and influence bone healing with active mineralization progressions on the implant surfaces. A greater thickness of the oxide layer on the surface is desirable because it increases the bone–implant contact [28] and reduces the bacterial biofilm [14]. Titanium develops an oxide layer when exposed to liquids or air, which reduces its reactivity and improves bone–implant contact [28]. The bioactive implant enhances the formation of hydroxyapatite on the surface of the implant in contact with body fluids [29]. TiO_2_ has these properties [30] and can be increased in thickness by thermal treatment [31], sol-gel coatings [32], and the physical-vapor-deposition (PVD) of titanium oxide [33]. Different techniques have been used to enhance the TiO_2_ layer, thermal, anodix [34], anodic oxidation [35], heat, enhancing bone formation, and improving initial stability [36,37,38]. Earlier research has demonstrated that this coating on the transmucosal part of the abutment minimizes the bacterial amount bound to the metal surface and creates healthy peri-implant tissues [39]. Also, a coating of the implant surface may be hypothesized, with beneficial results in cases of peri-implant crestal bone loss during peri-implant infection, when a coating reducing the bacterial load may have a positive impact in the treatment of peri-implantitis [40]. In the present study, a clean and not contaminated surface was found in the lasered group. In this research, all the specimens were similar in dimension and chemical surface structure but varied in surface topography and crystal structure as well as in surface oxide thickness. The results of this study demonstrated a significant increase of TO_2_ in specimens due to laser therapy. In a previous study, we found that the heat treatment of dental implants affected very primary events such as biomolecule adhesion, which may have an effect on cellular responses and the subsequent growth of tissue with the early formation of newly developed bone in direct contact with the surface of the implants [28]. Therefore, the improvement in bone–implant contact is attributed to the properties of the TiO_2_ layer. An in-vitro study with a primary culture of osteoblast cells showed that the modification of the implant surface does not alter but may enhance the excellent biocompatible comportment. Blood adhesion is usually favored on the surfaces treated with lasers [10].

## 5. Conclusions

In conclusion, these findings indicate that the laser-modified implant surfaces increase the thickness of TO_2_ and, due to clean processing surfaces, can be obtained with similar and repeatable characteristics. The clean surface can be clinically advantageous for reducing the healing period of the implant. This clean surface with the biological responses could be particularly advantageous for implants positioned in areas with very low-density bone, immediate loading requiring a bone regeneration procedure. Another advantage of a greater thickness of the oxide layer on the surface is that it may reduce the bacterial biofilm and increase the corrosion-resistant surfaces.

## Figures and Tables

**Table 1:** Atomic concentration of the elements detected on the machined and lasered implant surfaces from the X-Ray Photoelectron Spectroscopy (XPS) analysis.

Atomic Concentration %	[Ti]	[O]	[C]	[N]	[Al]	[Si]	[Ca]
Machined	16.0 ± 0.56	43.45 ± 1.62	34.6 ± 1.98	1.6 ± 0.28	3.85 ± 0.63	1.0 ± 0.4	-
Laser	16.4 ± 0.14	43.4 ± 0.28	36.25 ± 0.21	0.95 ± 0.07	-	1.4 ± 0.14	1.6 ± 0.28

## References

[1] Scarano A., Carinci F., Quaranta A., Iezzi G., Piattelli M., Piattelli A. (2008). Correlation between implant stability quotient (ISQ) with clinical and histological aspects of dental implants removed for mobility. Int. J. Immunopathol. Pharmacol..

[2] Burgos P.M., Rasmusson L., Meirelles L., Sennerby L. (2008). Early Bone Tissue Responses to Turned and Oxidized Implants in the Rabbit Tibia. Clin. Implant. Dent. Relat. Res..

[3] Larsson C., Thomsen P., Aronsson B.-O., Rodahl M., Lausmaa J., Kasemo B., Ericson L. (1996). Bone response to surface-modified titanium implants: studies on the early tissue response to machined and electropolished implants with different oxide thicknesses. Biomaterials.

[4] Scarano A., Degidi M., Perrotti V., Degidi D., Piattelli A., Iezzi G. (2013). Experimental Evaluation in Rabbits of the Effects of Thread Concavities in Bone Formation with Different Titanium Implant Surfaces. Clin. Implant. Dent. Relat. Res..

[5] Bowers B., Pollock K., Barclay S. (2020). Administration of end-of-life drugs by family caregivers during covid-19 pandemic. BMJ.

[6] Scarano A., Carinci F., Assenza B., Piattelli M., Murmura G., Piattelli A. (2011). Vertical ridge augmentation of atrophic posterior mandible using an inlay technique with a xenograft without miniscrews and miniplates: case series. Clin. Oral Implant. Res..

[7] Han C.-H., Johansson C.B., Wennerberg A., Albrektsson T. (1998). Quantitative and qualitative investigations of surface enlarged titanium and titanium alloy implants. Clin. Oral Implant. Res..

[8] Lausmaa J., Linder L. (1988). Surface spectroscopic characterization of titanium implants after separation from plastic-embedded tissue. Biomaterials.

[9] Okazaki Y., Gotoh E. (2005). Comparison of metal release from various metallic biomaterials in vitro. Biomaterials.

[10] Tengvall P., Lundström I. (1992). Physico-chemical considerations of titanium as a biomaterial. Clin. Mater..

[11] Wang G., Li J., Lv K., Zhang W., Ding X., Yang G., Liu X., Jiang X. (2016). Surface thermal oxidation on titanium implants to enhance osteogenic activity and in vivo osseointegration. Sci. Rep..

[12] Li L.-H., Kong Y.-M., Kim H.-W., Kim Y.-W., Kim H.-E., Heo S.-J., Koak J.-Y. (2004). Improved biological performance of Ti implants due to surface modification by micro-arc oxidation. Biomaterials.

[13] Lepore S., Milillo L., Trotta T., Castellani S., Porro C., Panaro M.A., Santarelli A., Bambini F., Muzio L.L., Conese M. (2013). Adhesion and growth of osteoblast-like cells on laser-engineered porous titanium surface: expression and localization of N-cadherin and beta-catenin. J. Boil. Regul. Homeost. Agents.

[14] Ionescu A.C., Brambilla E., Azzola F., Ottobelli M., Pellegrini G., Francetti L.A. (2018). Laser microtextured titanium implant surfaces reduce in vitro and in situ oral biofilm formation. PLOS ONE.

[15] Sinjari B., Traini T., Caputi S., Mortellaro C., Scarano A. (2018). Evaluation of Fibrin Clot Attachment on Titanium Laser-Conditioned Surface Using Scanning Electron Microscopy. J. Craniofac. Surg..

[16] Berardi D., De Benedittis S., Scoccia A., Perfetti G., Conti P. (2011). New laser-treated implant surfaces: a histologic and histomorphometric pilot study in rabbits. Clin. Investig. Med..

[17] Piattelli A. (2003). Residual aluminum oxide on the surface of titanium implants has no effect on osseointegration. Biomaterials.

[18] Wennerberg A., Albrektsson T., Lausmaa J. (1996). Torque and histomorphometric evaluation of c.p. titanium screws blasted with 25- and 75-microns-sized particles of Al_2_O_3_. J. Biomed. Mater. Res..

[19] Wennerberg A., Albrektsson T., Andersson B. (1995). An animal study of c.p. titanium screws with different surface topographies. J. Mater. Sci. Mater. Electron..

[20] Cao Y., Zeng X., Cai Z., Duan J. (2010). Laser micro/nano-fabrication techniques and their applications in electronics. Advances in Laser Materials Processing.

[21] Lee J.-T., Cho S.-A. (2016). Biomechanical evaluation of laser-etched Ti implant surfaces vs. chemically modified SLA Ti implant surfaces: Removal torque and resonance frequency analysis in rabbit tibias. J. Mech. Behav. Biomed. Mater..

[22] Inchingolo F., Ballini A., Cagiano R., Inchingolo A.D., Serafini M., De Benedittis M., Cortelazzi R., Tatullo M., Marrelli M., Inchingolo A.M. (2015). Immediately loaded dental implants bioactivated with platelet-rich plasma (PRP) placed in maxillary and mandibular region. La Clinica Terapeutica.

[23] Scarano A., Piattelli A., Quaranta A., Lorusso F. (2017). Bone Response to Two Dental Implants with Different Sandblasted/Acid-Etched Implant Surfaces: A Histological and Histomorphometrical Study in Rabbits. BioMed Res. Int..

[24] Ballo A.M., Bjöörn D., Åstrand M., Palmquist A., Lausmaa J., Thomsen P. (2012). Bone response to physical-vapour-deposited titanium dioxide coatings on titanium implants. Clin. Oral Implant. Res..

[25] Oh S., Tobin E., Yang Y., Carnes D.L., Ong J.L. (2005). In vivo evaluation of hydroxyapatite coatings of different crystallinities. Int. J. Oral Maxillofac. Implant..

[26] Marenzi G., Impero F., Scherillo F., Sammartino J.C., Squillace A., Spagnuolo G. (2019). Effect of Different Surface Treatments on Titanium Dental Implant Micro-Morphology. Materials.

[27] Ballo M.A., Omar O., Xia W., Palmquist A. (2011). Dental Implant Surfaces*—*Physicochemical Properties, Biological Performance, and Trends. Implant Dentistry—A Rapidly Evolving Practice.

[28] Scarano A., Crocetta E., Quaranta A., Lorusso F. (2018). Influence of the Thermal Treatment to Address a Better Osseointegration of Ti6Al4V Dental Implants: Histological and Histomorphometrical Study in a Rabbit Model. BioMed Res. Int..

[29] Kokubo T. (2005). Design of bioactive bone substitutes based on biomineralization process. Mater. Sci. Eng. C.

[30] Kokubo T., Kushitani H., Sakka S., Kitsugi T., Yamamuro T. (1990). Solutions able to reproducein vivo surface-structure changes in bioactive glass-ceramic A-W3. J. Biomed. Mater. Res..

[31] Wang X.-X., Hayakawa S., Tsuru K., Osaka A. (2000). Improvement of bioactivity of H_2_O_2_/TaCl_5_-treated titanium after subsequent heat treatments. J. Biomed. Mater. Res..

[32] Rossi S., Moritz N., Tirri T., Peltola T., Areva S., Jokinen M., Happonen R.-P., Närhi T. (2007). Comparison between sol-gel-derived anatase- and rutile-structured TiO_2_ coatings in soft-tissue environment. J. Biomed. Mater. Res. Part A.

[33] Zhou W., Zhong X., Wu X., Yuan L., Shu Q., Xia Y., Ostrikov K. (2007). (Ken) Plasma-controlled nanocrystallinity and phase composition of TiO_2_: A smart way to enhance biomimetic response. J. Biomed. Mater. Res. Part A.

[34] Chen G., Wen X., Zhang N. (1998). Corrosion resistance and ion dissolution of titanium with different surface microroughness. Bio-Med. Mater. Eng..

[35] Ishizawa H., Ogino M. (1995). Formation and characterization of anodic titanium oxide films containing Ca and P. J. Biomed. Mater. Res..

[36] Park I.S., Yang E.J., Bae T.S. (2013). Effect of Cyclic Precalcification of Nanotubular TiO_2_ Layer on the Bioactivity of Titanium Implant. BioMed Res. Int..

[37] Nguyen T.-D.T., Park I.-S., Lee M.-H., Bae T.-S. (2013). Enhanced biocompatibility of a pre-calcified nanotubular TiO_2_ layer on Ti–6Al–7Nb alloy. Surf. Coat. Technol..

[38] Butt A., Hamlekhan A., Patel S.B., Royhman D., Sukotjo C., Mathew M.T., Shokuhfar T., Takoudis C. (2015). A Novel Investigation of the Formation of Titanium Oxide Nanotubes on Thermally Formed Oxide of Ti-6Al-4V. J. Oral Implant..

[39] Scarano A., Piattelli A., Polimeni A., Di Iorio D., Carinci F. (2010). Bacterial Adhesion on Commercially Pure Titanium and Anatase-Coated Titanium Healing Screws: An In Vivo Human Study. J. Periodontol..

[40] Ballini A., Cantore S., Farronato D., Cirulli N., Inchingolo F., Papa F., Malcangi G., Inchingolo A.D., DiPalma G., Sardaro N. (2015). Periodontal disease and bone pathogenesis: The crosstalk between cytokines and porphyromonas gingivalis. J. Boil. Regul. Homeost. Agents.

